# Protective Effects of Cinnamaldehyde on the Inflammatory Response, Oxidative Stress, and Apoptosis in Liver of *Salmonella typhimurium*-Challenged Mice

**DOI:** 10.3390/molecules26082309

**Published:** 2021-04-16

**Authors:** Renjie Wang, Senlin Li, Hai Jia, Xuemeng Si, Yan Lei, Jirong Lyu, Zhaolai Dai, Zhenlong Wu

**Affiliations:** 1 State Key Laboratory of Animal Nutrition, China Agricultural University, Beijing 100193, China; wangrenjie@cau.edu.cn (R.W.); smartfish2017@cau.edu.cn (S.L.); jiahai@cau.edu.cn (H.J.); sisi_sxm@cau.edu.cn (X.S.); daizhaolai@cau.edu.cn (Z.D.); 2 DadHank Biotechnology Corporation, Chengdu 611130, China; leiyantyp413@163.com (Y.L.); lvjirong468@126.com (J.L.)

**Keywords:** cinnamaldehyde, *Salmonella typhimurium*, mice, inflammation, apoptosis, oxidative stress, gut microbiota

## Abstract

*Salmonella typhimurium* infection is associated with gastrointestinal disorder and cellular injury in the liver of both humans and animals. Cinnamaldehyde, the main component of essential oil from cinnamon, has been reported to have anti-inflammatory, anti-oxidative, and anti-apoptotic effects. However, it remains unknown whether cinnamaldehyde can alleviate *Salmonella typhimurium* infection-induced liver injury in mice. In the present study, we found that cinnamaldehyde attenuated *Salmonella typhimurium*-induced body weight loss, the increase of organ (liver and spleen) indexes, hepatocyte apoptosis, and the mortality rate in mice. Further study showed that cinnamaldehyde significantly alleviated *Salmonella typhimurium*-induced liver injury as shown by activities of alanine transaminase, aspartate transaminase, and myeloperoxidase, as well as malondialdehyde. The increased mRNA level of pro-inflammatory cytokines (*IL-1β*, *IL-6*, *TNF-α*, and *IFN-γ*) and chemokines (*CCL2* and *CCL3*) induced by *Salmonella typhimurium* were significantly abolished by cinnamaldehyde supplementation. These alterations were associated with a regulatory effect of cinnamaldehyde on *TLR2*, *TLR4*, and *MyD88*. 16S rDNA sequence analysis showed that *Salmonella typhimurium* infection led to upregulation of the abundances of genera *Akkermansia*, *Bacteroides*, *Alistipes*, *Muribaculum*, and *Prevotellaceae UCG-001*, and downregulation of the abundances of genera *Lactobacillus*, *Enterorhabdus*, and *Eggerthellaceae* (unclassified). These alterations were reversed by cinnamaldehyde supplementation. In conclusion, cinnamaldehyde attenuated the inflammatory response, oxidative stress, and apoptosis in the liver of *Salmonella typhimurium*-infected mice. Supplementation of cinnamaldehyde might be a preventive strategy to alleviate liver injury caused by *Salmonella typhimurium* infection in humans and animals.

## 1. Introduction

The liver plays critical roles in various physiological activities, such as the metabolism of nutrients, detoxification, and immune responses [1]. A variety of factors, including alcohol abuse, drug exposure and pathogens infection have been reported to be associated with liver injury in humans [2]. *Salmonella typhimurium* is one of the primary foodborne pathogens, whose infection has been observed in both humans and animals all over the world [3]. After entering the gut following ingestion, *Salmonellae* reaches the intestinal epithelium and impairs the intestinal barrier function [4], leads to intestinal dysbiosis [5], and triggers the development of the gastrointestinal disease. *Salmonellae* infection is accompanied by injury of other tissues, including the liver and spleen [6,7], as shown by inflammatory cell infiltration, intense congestion, hepatic apoptosis, and oxidative damage due to the complicated interactions between the gut and the liver through the gut-liver axis [8,9,10]. Cytokines (interleukin-1β (IL-1β), interleukin-6 (IL-6), tumor necrosis factor-α (TNF-α) and interferon γ (IFN-γ)) are primarily derived from mononuclear phagocytic cells and other antigen-presenting cells, which play an important role in promoting the cellular infiltrate and damage to resident tissue characteristic of inflammation [11]. Chemokines (C–C motif chemokine ligand 2 (CCL2) and CCL3) are produced at the site of infection or in response to a proinflammatory stimulus, recruiting and activating leukocytes to promote the immune response [11]. *Salmonella typhimurium* infection always results in strong inflammatory response and accompanied by secretion of cytokines and chemokines and leading inflammatory damage [12,13,14].

Natural products have gained increasing attention due to bacteriostatic and anti-inflammatory activity [4,15,16]. Cinnamaldehyde is the key active component of cinnamon oil extracted from cinnamon trees and other species of the genus *Cinnamomum* [17]. Numerous studies have been reported that cinnamaldehyde possesses anti-bacterial, anti-inflammatory, and anti-oxidative properties. In vitro studies showed that cinnamaldehyde attenuated lipopolysaccharide (LPS)-stimulated activation of the immune response in macrophages [18] and alleviated H_2_O_2_-induced oxidative stress through the nuclear factor erythroid 2 (NF-E2) p45-related factor 2 (NRF2) and heme oxygenase-1 (HO-1)-dependent antioxidant response in human dental pulp cells [19]. In the mouse model, cinnamaldehyde mitigated intestinal inflammation-induced by *Cronobacter sakazakii* through suppressing *IL-1β*, *IL-6*, *TNF-α* expression, inhibiting the activation of nuclear factor-kappa B (NF-κB) signaling pathway, and reduced apoptosis by inhibiting caspase3 activity in enterocyte [20]. In a recent study, the authors revealed that cinnamaldehyde inhibited the expression of *Salmonella* pathogenicity island 1 (SPI-1), an effector protein, therefore decreasing colonization of *Salmonella* and improving the intestinal barrier function of mice [15]. Despite these observations, it remains unknown whether cinnamaldehyde can alleviate *Salmonella typhimurium*-induced liver injury. In the present study, mice pretreated with cinnamaldehyde were infected with *Salmonella typhimurium*. Oxidative stress, mRNA level of inflammatory cytokines, apoptosis of hepatocytes, as well as colonic microbiota were analyzed.

## 2. Results

### 2.1. The Protective Effect of Cinnamaldehyde on the Feed Intake, Body Weight Change, and Organ Index of Salmonella typhimurium-Infected Mice

**Figure 1:**
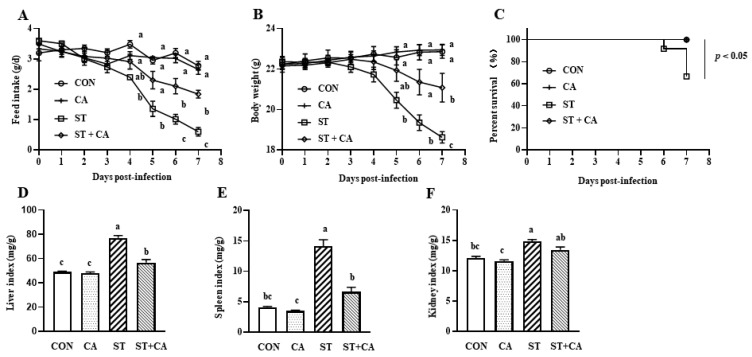
Cinnamaldehyde attenuated the *Salmonella typhimurium*-infected body weight loss and mortality. (**A**) Feed intake (*n* = 4); (**B**) body weight change (*n* = 8–12); (**C**) survival rate; (**D**) liver index; (**E**) spleen index; (**F**) kidney index of C57BL/6J mice. Data are shown as mean ± SEM. Means without a common letter differ (*p* < 0.05). CON, control group, CA, cinnamaldehyde group; ST, *Salmonella typhimurium SL1344* group; ST + CA, *Salmonella typhimurium SL1344* + cinnamaldehyde group.

As shown in Figure 1A,B, feed intake and body weight of mice in the *Salmonella typhimurium* (ST) group were less than that of the control (CON) group on day 7 after *Salmonella typhimurium* challenge (*p* < 0.05). These effects of *Salmonella typhimurium* were significantly abrogated by cinnamaldehyde (*p* < 0.05). Cinnamaldehyde supplementation had no effect on feed intake and body weight of mice, as compared with the CON group. No mice died in the CON, cinnamaldehyde (CA), or *Salmonella typhimurium SL1344* + cinnamaldehyde (ST + CA) groups during the entire experimental time. However, 33.33% of the mice in the ST group did not survive (Figure 1C). The organ (liver, spleen, and kidney) index of mice in the ST treatment group were increased as compared with these of mice in the CON group (*p* < 0.05) (Figure 1D–F). The increased organ index of liver and spleen, instead of kidney, following *Salmonella typhimurium* infection were alleviated by cinnamaldehyde administration (*p* < 0.05).

### 2.2. Cinnamaldehyde Alleviated the Salmonella typhimurium-Induced Liver Damage

**Figure 2:**
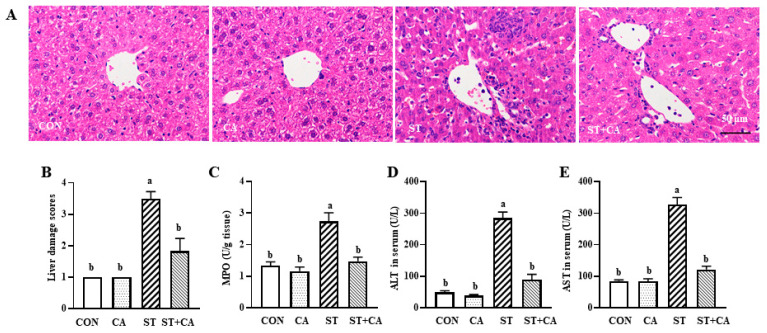
Cinnamaldehyde attenuated the *Salmonella typhimurium*-induced histopathological alterations in liver tissues. (**A**) Histological observation, (**B**) Liver damage scores, (**C**) MPO, (**D**) ALT, and (**E**) AST were analyzed on day 7 after *Salmonella typhimurium*-infection. Values are expressed as mean ± SEM, *n* = 6 in each group. Means without a common letter differ (*p* < 0.05). Scale bar, 50 μm. CON, control group, CA, cinnamaldehyde group; ST, *Salmonella typhimurium SL1344* group; ST + CA, *Salmonella typhimurium SL1344* + cinnamaldehyde group.

Compared with the CON group, *Salmonella typhimurium* infection significantly increased (*p* < 0.05) infiltration of inflammatory cells, hemorrhage, and damage score in the liver of mice (Figure 2A,B). These changes were attenuated by cinnamaldehyde supplementation (*p* < 0.05). Consistently, the activities of myeloperoxidase (MPO) in liver tissue, aspartate aminotransferase (AST) and alanine transaminase (ALT) in serum were enhanced (*p* < 0.05) by the *Salmonella typhimurium* challenge, and these effects were abrogated by cinnamaldehyde addition (Figure 2C–E). Compared with the CON group, only cinnamaldehyde supplementation had no effect on damage score, MPO, AST, and ALT of mice.

### 2.3. The Effect of Cinnamaldehyde and Salmonella typhimurium on the Expression of Inflammation-Related Genes in the Liver

**Figure 3:**
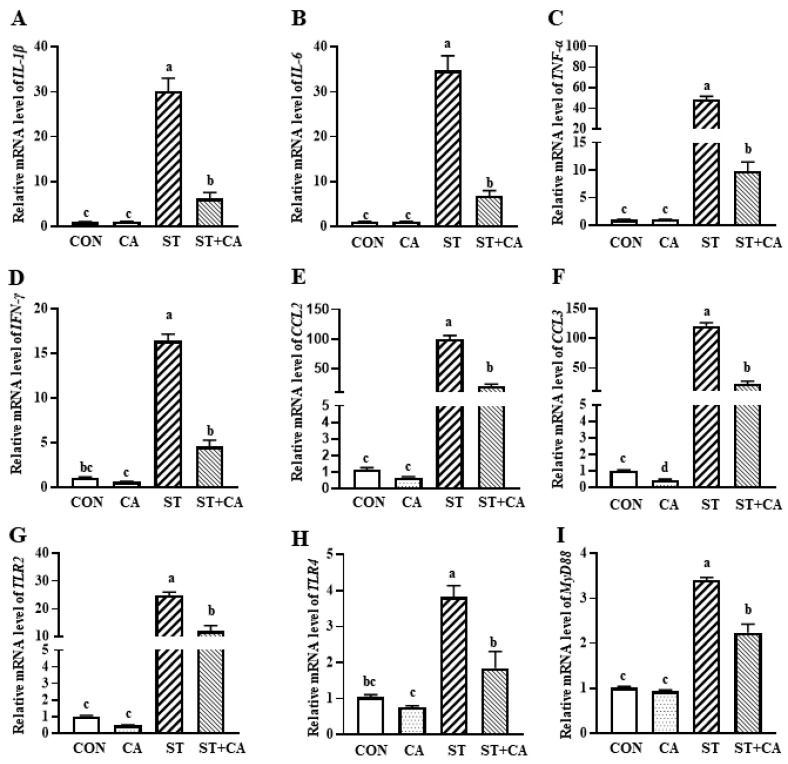
The effect of cinnamaldehyde and/or *Salmonella typhimurium* on the mRNA level of inflammation-related genes in the liver. (**A**) *IL-1β*, (**B**) *IL-6*, (**C**) *TNF-α*, (**D**) *IFN-γ*, (**E**) *CCL2*, (**F**) *CCL3*, (**G**) *TLR2*, (**H**) *TLR4*, and (**I**) *MyD88* mRNA level in the liver. Values are expressed as mean ± SEM, *n* = 6 in each group. Means without a common letter differ (*p* < 0.05). CON, control group, CA, cinnamaldehyde group; ST, *Salmonella typhimurium SL1344* group; ST + CA, *Salmonella typhimurium SL1344* + cinnamaldehyde group.

Real-time PCR analysis showed that *Salmonella typhimurium* challenge increased (*p* < 0.05) mRNA expression of proinflammatory cytokines (*IL-1β*, *IL-6*, *TNF-α*, and *IFN-γ*) and chemokines (*CCL2* and *CCL3*), while cinnamaldehyde supplementation alleviated (*p* < 0.05) these effects (Figure 3A–F). Only cinnamaldehyde supplementation down-regulated the CCL3 expression (*p* < 0.05), as compared with CON group. Besides, the mRNA expression of Toll-like receptors (*TLRs*) and myeloid differentiation factor 88 (*MyD88*) were determined. Compared with CON, *Salmonella typhimurium* infection enhanced (*p* < 0.05) mRNA expression of *TLR2*, *TLR4*, and *MyD88*. Interestingly, cinnamaldehyde supplementation prevented (*p* < 0.05) these effects of *Salmonella typhimurium* infection (Figure 3G–I).

### 2.4. Cinnamaldehyde Attenuated Salmonella typhimurium-Induced Hepatic Apoptosis in Mice

**Figure 4:**
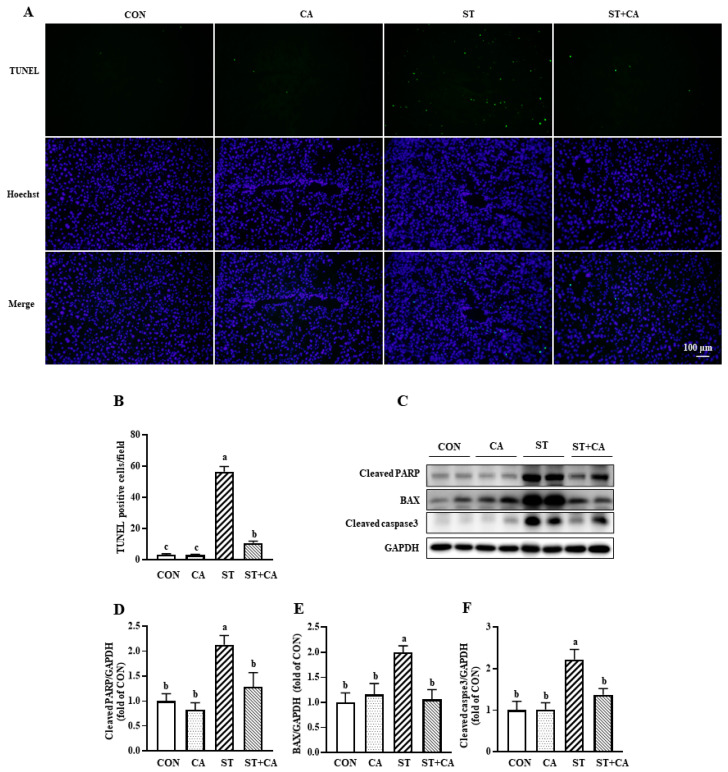
Cinnamaldehyde attenuated *Salmonella typhimurium*-induced hepatic apoptosis in mice. (**A**) TUNEL assay, scale bar, 100 μm; (**B**) TUNEL-positive cells in the liver. (**C**) Representative protein bands of cleaved PARP, BAX, cleaved capase3, and GAPDH; (**D**–**F**) Statistical analysis of protein abundance. Values are expressed as mean ± SEM, *n* = 6 in each group. Means without a common letter differ (*p* < 0.05). CON, control group, CA, cinnamaldehyde group; ST, *Salmonella typhimurium SL1344* group; ST + CA, *Salmonella typhimurium SL1344* + cinnamaldehyde group.

To investigate whether the protective effects of cinnamaldehyde on liver damage were associated with reduced apoptosis of hepatic cells, Terminal deoxynucleotidyl transferase-mediated dUTP nick end labeling (TUNEL) assay was performed and the amount of TUNEL positive cells were counted. As illustrated, the *Salmonella typhimurium* challenge resulted in enhanced (*p* < 0.05) apoptosis, which was alleviated (*p* < 0.05) by cinnamaldehyde supplementation (Figure 4A,B). Consistent with the TUNEL analysis results, the protein level of cleaved poly (ADP-ribose) polymerase (PARP), B cell lymphoma 2-associated X (BAX), and cleaved caspase3 were increased (*p* < 0.05) in the *Salmonella typhimurium* challenge, which were significantly decreased (*p* < 0.05) by cinnamaldehyde supplementation (Figure 4C–F).

### 2.5. Cinnamaldehyde Attenuated Hepatic Oxidative Stress in Salmonella typhimurium-Infected Mice

**Figure 5:**
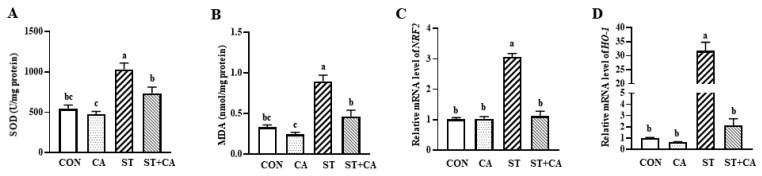
Cinnamaldehyde attenuated hepatic oxidative stress in *Salmonella typhimurium*-infected mice. (**A**) Superoxide dismutase (SOD) and (**B**) malondialdehyde (MDA) level in the liver, (**C**) *NRF2*, (**D**) *HO-1* mRNA level in the liver. Values are expressed as mean ± SEM, *n* = 6 in each group. Means without a common letter differ (*p* < 0.05). CON, control group, CA, cinnamaldehyde group; ST, *Salmonella typhimurium SL1344* group; ST + CA, *Salmonella typhimurium SL1344* + cinnamaldehyde group.

Compared with the CON group, *Salmonella typhimurium* treatment increased (*p* < 0.05) hepatic activity of superoxide dismutase (SOD) and the concentration of malondialdehyde (MDA), which were attenuated by cinnamaldehyde supplementation (Figure 5A,B). The hepatic mRNA level of NRF2 and HO-1 were also analyzed. *Salmonella typhimurium* treatment enhanced (*p* < 0.05) the NRF2 and HO-1 mRNA levels, and cinnamaldehyde addition eliminated (*p* < 0.05) these effects of *Salmonella typhimurium* infection (Figure 5C,D).

### 2.6. Effect of Cinnamaldehyde and Salmonella typhimurium on the Microbiota Community in Colonic Content

**Figure 6:**
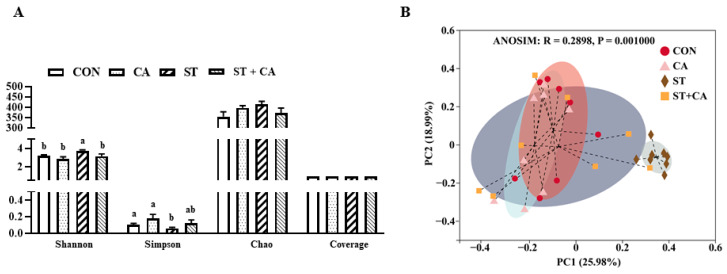
Effect of cinnamaldehyde and *Salmonella typhimurium* on alpha and beta diversity of microbiota in colonic content. (**A**) alpha diversity and (**B**) beta diversity. Values are expressed as mean ± SEM, *n* = 8 in each group. Means without a common letter differ (*p* < 0.05). CON, control group, CA, cinnamaldehyde group; ST, *Salmonella typhimurium SL1344* group; ST + CA, *Salmonella typhimurium SL1344* + cinnamaldehyde group.

Compared with the CON group, the Shannon index enhanced (*p* < 0.05) and Simpson index decreased (*p* < 0.05) after *Salmonella typhimurium* infection, which were attenuated by cinnamaldehyde supplementation. However, there were no significant effect (*p* > 0.05) of cinnamaldehyde or *Salmonella typhimurium* on Chao and Coverage indexes (Figure 6A). To evaluate overall differences in beta-diversity, principal coordinate analysis (PCoA) was used to identify discrepancies among groups. PCoA was conducted based on Bray–Curtis distance of operational taxonomic unit (OTU) relative abundance in colonic content. Objects that are ordinated closer together have smaller dissimilarity values than those ordinated further apart. As shown in Figure 6B, the ST group was separated from CON, CA, and ST + CA groups (ANOSIM: R = 0.29, *p* < 0.05). which means *Salmonella typhimurium* infection changed the bacteria community structure, but cinnamaldehyde treatment reversed this effect of *Salmonella typhimurium* challenge.

**Figure 7:**
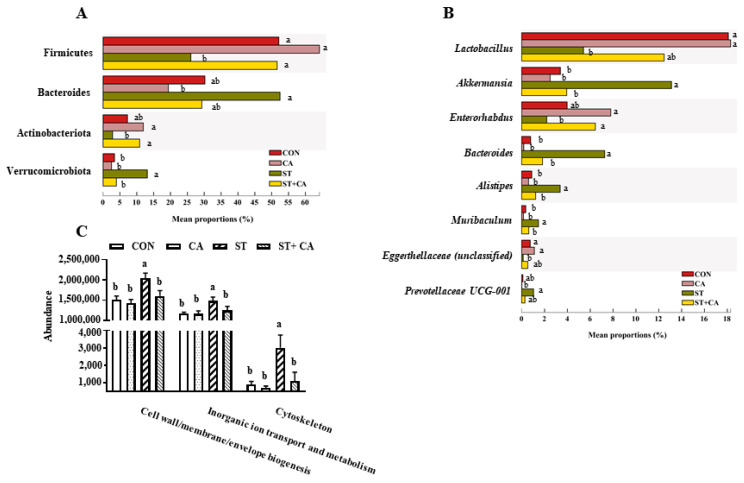
Effect of cinnamaldehyde and *Salmonella typhimurium* on the microbiota community in colonic content. The difference of colonic microbiota at (**A**) phylum level, (**B**) genus level, and (**C**) COG category in each group. Values are expressed as mean ± SEM, *n* = 8 in each group. Means without a common letter differ (*p* < 0.05). CON, control group, CA, cinnamaldehyde group; ST, *Salmonella typhimurium SL1344* group; ST + CA, *Salmonella typhimurium SL1344* + cinnamaldehyde group.

At the phylum level, compared with the CON group, *Salmonella typhimurium* infection enhanced (*p* < 0.05) the abundance of *Bacteroidota* and *Verrumicrobiota*, decreased (*p* < 0.05) the levels of *Firmicutes* and *Actinobacteriota*, but these effects were attenuated by cinnamaldehyde addition (Figure 7A). As shown in Figure 7B, compared with the CON group, the abundances of genera *Akkermansia*, *Bacteroides*, *Alistipes*, *Muribaculum*, and *Prevotellaceae UCG-001* were upregulated (*p* < 0.05), and the abundances of genera *Lactobacillus*, *Enterorhabdus*, and *Eggerthellaceae* (unclassified) were downregulated (*p* < 0.05) in ST group. While these changes in the bacteria community induced by *Salmonella typhimurium* were eliminated or attenuated by cinnamaldehyde supplementation.

Functional analyses based on the occurrence of clusters of orthologous groups (COGs) of proteins (Figure 7C) showed that the *Salmonella typhimurium* challenge resulted in a statistical increase in the abundance of “cell wall/membrane/envelope biogenesis”, “inorganic ion transport and metabolism”, and “cytoskeleton”. However, cinnamaldehyde supplementation reversed these effects of the *Salmonella typhimurium* challenge.

## 3. Discussion

In the present study, we found that *Salmonella typhimurium* infection-resulted in body weight loss, decreased feed intake, as well as increased mortality and organ index of liver and spleen, which were abrogated by cinnamaldehyde administration, indicating a protective effect of cinnamaldehyde against *Salmonella typhimurium*. This effect was verified by histological alteration as shown by H&E staining. ALT and AST are well-known biomarkers for tissue injury or inflammation [21]. Consistent with previous studies [4,22,23], *Salmonella typhimurium* infection resulted in a dramatic increase of ALT and AST levels in serum, as well as increased MPO activity, an indicator of increased number of neutrophilic granulocytes. Interestingly, the adverse effects of *Salmonella typhimurium* infection were abrogated by cinnamaldehyde, indicating a reduction in hepatic injury and neutrophil infiltration.

Biosynthesis and secretion of pro-inflammatory cytokines and chemokines by macrophage is associated with hepatic injury in animals [2,24,25,26]. To test the implication of inflammatory cytokines and their contribution to the beneficial effect of cinnamaldehyde, we analyzed the expression of pro-inflammatory cytokines and chemokines in the liver. As expected, the enhanced mRNA level of pro-inflammatory cytokines (*IL-1β*, *IL-6*, *TNF-α*, and *IFN-γ*) and chemokines (*CCL2* and *CCL3*) in response to *Salmonella typhimurium* infection were remarkably reduced by cinnamaldehyde supplementation. TLRs are responsible for the recognition of pathogen-associated molecular patterns (PAMPs) during pathogen infection, such as TLR2 recognizing lipoproteins and glycolipids and TLR4 recognizing LPS [27]. Activation of TLR2 and TLR4 promotes the release of pro-inflammatory cytokine and chemokine in MyD88-dependent pathways [28,29]. Several reports have revealed the involvement of TLR2 and TLR4 in *Salmonella typhimurium* infected mouse [4,30,31]. In agreement with previous studies, we observed the upregulation of *TLR2*, *TLR4*, and *MyD88* following, which was also attenuated by cinnamaldehyde addition. This result indicated that cinnamaldehyde could attenuate hepatic inflammatory injury caused by *Salmonella typhimurium* infection via MyD88-dependent TLR2/TLR4 signaling.

Accumulating evidence has shown that oxidative stress-related apoptosis is involved in pathogens infection-induced tissue injury [32,33,34]. We next evaluated apoptosis in the liver of mice using the TUNEL assay [35]. As expected, *Salmonella typhimurium* infection led to increased apoptosis, as well as enhanced protein expression of pro-apoptotic proteins, such as BAX, cleaved PARP, and cleaved caspase3 [2,36]. These effects triggered by *Salmonella typhimurium* infection were abolished by cinnamaldehyde administration.

NRF2, a redox sensitive transcription factor, locates in the cytoplasm under normal conditions [37]. In response to oxidative stress, NRF2 is activated and translocated into the nucleus, therefore activating the expression of genes implicated in antioxidant response and enzymes related to detoxification, such as SOD and HO-1 [37]. MDA is a biomarker of lipid peroxidation and oxidative stress in response to various pathogenic bacteria challenges [38]. In consistent with a previous study, we observed increased concentration of MDA in the liver tissues, increased activity of SOD, and elevated mRNA expression of *NRF2* and *HO-1* in the liver of *Salmonella typhimurium* challenged mice [39], indicating enhanced antioxidant activities to bacterial infection [40]. Cinnamaldehyde supplementation abrogated these alterations induced by *Salmonella typhimurium* infection, therefore contributing to alleviated the oxidative stress.

As one of the most prevalent foodborne pathogens, *Salmonella typhimurium* infection is associated with impairment of the intestinal barrier and development of the gastrointestinal disease, in which intestinal microbiota is involved [5,41,42,43]. Several bacteria, such as *Akkermansia* [44,45], *Bacteroides* [46], *Alistipes* [47], *Prevotellaceae UCG-001* [48], have been reported to be associated with intestinal barrier dysfunction and intestinal inflammation. In contrast, probiotics, such as *Lactobacillus*, with the ability to regulate intestinal bacteria of the host has shown improved intestinal barrier and reduced tissue injury [5,49]. In the present study, we found that *Salmonella typhimurium* infection resulted in the enhanced abundances of genera *Akkermansia*, *Bacteroides*, and *Alistipes*, and the reduced abundances of genera *Lactobacillus*, *Enterorhabdus*, and *Eggerthellaceae* (unclassified), which was reversed by cinnamaldehyde supplementation. This beneficial effect might be due to a complicated mechanism. First, cinnamaldehyde could penetrate the cell envelope and disrupt the structure of *Salmonella typhimurium* cells, therefore leading to reduced proliferation of bacteria [50]. Second, invasion of host cells relies on *Salmonella* pathogenicity Island 1 (SPI-1) proteins, a regulator responsible for cytoskeleton remodeling of the host cell in response to pathogen exposure. Cinnamaldehyde inhibits the expression of SPI-1 and leads to reduced invasion of the bacteria into host cells [15]. Third, cinnamaldehyde might regulate the formation of biofilm of *Salmonella typhimurium* and contributes to improving barrier function [51,52]. Further studies are needed to explore which mechanisms are the predominant contributor in our animal model.

Taken together, we demonstrated that cinnamaldehyde attenuated *Salmonella typhimurium*-induced mouse liver injury. The beneficial effect of cinnamaldehyde was associated with reduced inflammatory response, decreased hepatic apoptosis, and restoration of intestinal barrier gut microbiota. Supplementation of cinnamaldehyde might be a preventive strategy to alleviate liver injury caused by *Salmonella typhimurium* infection in humans and animals.

## 4. Materials and Methods

### 4.1. Materials

Male C57BL/6 mice were 6–8 weeks old and purchased from Huafukang Biotechnology Ltd. (Beijing, China). Cinnamaldehyde was purchased from Sigma Chemical Co. (St. Louis, MI, USA). Sodium carboxymethylcellulose was obtained from Sinopharm Chemical Reagent Co., Ltd. (Shanghai, China). *Salmonella typhimurium SL1344* was kindly gifted from professor Junjun Wang, China Agricultural University, China. ALT, AST, MPO, SOD, and MDA assay kits were purchased from Nanjing Jiancheng Bioengineering Institute (Nanjing, China). TUNEL apoptosis detection kit was procured from Beyotime Biotechnology (Beijing, China). Antibodies against PARP cleavage, BAX, and cleaved caspase3 antibody were obtained from Cell Signaling Technology (Beverly, MA, USA). Anti-GAPDH antibody was purchased from Santa Cruz Biotechnology Inc. (Santa Cruz, CA, USA). Horseradish peroxidase (HRP)-conjugated goat anti-rabbit secondary antibodies were purchased from Sangon Biotech Co. (Shanghai, China). An enhanced chemiluminescence kit was obtained from Applygen Technology Inc. (Beijing, China). TRIzol reagent was purchased from CWBIO Biotechnology Co. (Beijing, China). The first-strand cDNA synthesis kit was purchased from Tiangen Biotechnology Co. (Beijing, China). SYBR Green real-time PCR master mix was procured from Yeasen Biotechnology Co. (Shanghai, China). Primers were synthesized by Sangon Biotech Co. Hematoxylin & eosin (H&E) kit was the product of ZSGB Biotechnology (Beijing, China).

### 4.2. Culture of Bacteria

*Salmonella typhimurium SL1344* was cultured in LB media at 37 °C and 5% CO_2_ overnight, and the quantity of bacteria was counted. Bacteria were harvested by centrifugation at 12,000 rpm for 5 min, resuspended in sterile phosphate buffered saline (PBS) and used for the next experiment.

### 4.3. Animal Experiments

All experimental procedures were approved by the Institutional Animal Care and Use Committee of China Agricultural University (AW40101202-1-1). A total of 48 male C57BL/6 mice were housed in a standard housing condition (temperature, 25 ± 2 °C; relative humidity, 50 ± 5%; lighting cycle, 12 h/day). Mice had free access to feed (Huafukang Biotechnology Ltd., Beijing, China) and drinking water. After one week of adaptive feeding, the mice were randomly assigned into 4 groups (*n* = 12 per group): Control (CON), Cinnamaldehyde (CA), *Salmonella typhimurium SL1344* (ST), *Salmonella typhimurium SL1344* + Cinnamaldehyde (ST + CA). Cinnamaldehyde dissolved in 0.5% sodium carboxymethylcellulose (CMC-Na). ST + CA and CA groups received an intragastric administration of cinnamaldehyde (40 mg/kg body weight) once a day for 14 consecutive days, and the CON and ST groups were received equal volumes of 0.5% CMC-Na. On day 7, the mice were orally infected with 0.2 mL of PBS containing with or without 5.0 × 10^6^ CFU/mL *Salmonella typhimurium*. On day 14, blood was taken from the retroorbital sinus, and the liver, spleen, and colonic content samples were collected for further analysis after mice were killed by cervical dislocation. Serum samples were obtained from blood samples centrifuged at 3000 rpm for 15 min at 4 °C. All samples were immediately obtained and stored at −80 °C until analysis.

### 4.4. Biochemical Index Estimation

The level of ALT and AST in serum were measured by using commercial kits according to the manufacturer’s instructions (Nanjing Jiancheng Bioengineering Institute, Nanjing, China). Liver homogenates were used for the detection of the activity of enzymes (MPO and SOD), and the concentration of MDA by spectrophotometric methods, as instructed by the manufacturer. Data on hepatic MDA content, as well as SOD activity are expressed per milligram of protein, whereas data on hepatic MPO activity was expressed per gram of tissue.

### 4.5. Histological Analysis

Liver tissues embedded in the O.C.T compound were sectioned and stained with H&E. Sections from each mouse (*n* = 6) were visualized using a light microscope equipped with a computer-assisted morphometric system at a magnification of 400 by a blinded observer. 4 fields in each section were examined and representative images were provided. To evaluate the degree of liver damage, an injury grading score (Grade 1–4) based on the amount of infiltration of inflammatory cells, hemorrhage, and hepatocyte necrosis was carried out following a previous report [53]. The degree of liver damage was estimated by the following criteria: Grade 1, 0 to 25% of damage; Grade 2, 25% to 50% of damage; Grade 3, 50% to 75% of damage; Grade 4, 75% to 100% of damage.

### 4.6. TUNEL Assay

Cell apoptosis was detected by TUNEL assay according to the manufacturer’s instructions (Beyotime Biotech, Beijing, China). Sections of the liver tissues were permeabilized with 0.5% Triton X-100 for 5 min at room temperature, followed by incubation with a TUNEL reaction mixture for 1 h at 37 °C. Nuclei were stained with Hoechst 33342 (10 μg·mL^−1^) for 2 min at room temperature. Sections from each mouse (*n* = 3) were observed by a blinded observer under a fluorescence microscope (Axio Vert.A1, Zeiss, Jena, Germany). The TUNEL positive cells in 2 randomly selected fields of each section were counted.

### 4.7. Quantitative Real-Time PCR (qRT-PCR)

Total RNA was extracted from liver tissues using TRIzol reagent according to the manufacturer’s protocol. RNA concentration and value of OD260/280 were measured using the Nanodrop P330 (Implen, Munich, Germany). The integrity of total RNA was assessed by 1% agarose gel electrophoresis. Reverse-transcription was performed according to the manufacturer’s instruction. qRT-PCR was performed on the ABI-Prism 7500 Sequence Detection System (Applied Biosystems, Foster City, CA, USA) using the SYBR Green real-time master mix. The primer sequences of genes used in the present study are listed in Table 1. Gene expression levels were normalized with GAPDH transcripts based on the 2^−ΔΔCt^ method.

### 4.8. Western Blot Analysis

Liver tissues were dissolved and vortexed in cold RIPA buffer (10 mm Tris-HCl, pH 7.4; 150 mm NaCl; 10 mm EDTA; 1% NP-40; 0.1% SDS) supplemented with protease and phosphatase inhibitors. After centrifugation, the supernatant fluid of the dissolved tissue was measured by the BCA method. Protein abundance was determined using the Western blot technique, as reported previously [54]. Protein bands were visualized by an enhanced chemiluminescence kit using the ImageQuant LAS 4000 mini system (GE Healthcare, Piscataway, NJ, USA).

### 4.9. 16S rDNA Sequencing

Microbial DNA of colonic content samples were extracted using the E.Z.N.A.^®^ Soil DNA Kit (Omega Bio-tek, Norcross, GA, USA) according to the manufacturer’s protocol. The quality of DNA was checked by 1% agarose gel electrophoresis, and DNA concentration was measured by using a Nanodrop (Thermo Scientific, Wilmington, DE, USA). The V3–V4 gene region of 16S rRNA was amplified by using the primers 338F and 806R by an ABI GeneAmp^®^ 9700 PCR thermocycler (ABI, Vernon, CA, USA). The amplicons were then extracted from 2% agarose gels and further purified using the AxyPrep DNA Gel Extraction Kit (Axygen Biosciences, Union City, CA, USA) and quantified by Quantus™ Fluorometer (Promega, Madison, WI, USA). Purified amplicons were pooled in equimolar and paired-end sequencing on an Illumina MiSeq platform (Illumina, San Diego, CA, USA) by Shanghai Majorbio Bio-Pharm Technology Co., Ltd. (Shanghai, China). Sequences were quality filtered and clustered into OTUs at 97% identity [55]. The sequencing data generated in this study have been submitted to the NCBI Sequence Read Archive (BioProject: PRJNA698466).

### 4.10. Statistical Analysis

Data are presented as means ± SEMs and were tested for normality and homoscedasticity using the Kolmogorov-Smirnov and Levene’s tests (with the significance level set at 5%). Differences were analyzed by SPSS 25.0 statistical software (SPSS, Chicago, IL, USA) with the Kruskal–Wallis test or one-way ANOVA followed by Tukey’s post hoc test. Survival analyses was used Log Rank (Mantel-Cox) test. For the analysis of bio-information, phylum, and genus at < 1.0% relative abundance were excluded from all analyzes. Alpha diversity (Shannon, Simpson, Chao, and Coverage) was assessed by Mothur (Version 1.35.0) [56]. PCoA was conducted based on Bray–Curtis distance of OTUs relative abundance in colonic content microbiota. The differences between groups at the phylum and genus levels were analyzed by the Kruskal–Wallis test. Graphs were generated by Prism 8 (2018 GraphPad Software, Inc., La Jolla, CA, USA). *p* < 0.05 was considered as statistically significant.

## Figures and Tables

**Table 1:** Primer sequences used in qRT-PCR.

Gene	Forward Primer (5′to 3′)	Reverse Primer (5′to 3′)
*IL-1β*	TGCACTACAGGCTCCG	TGCCGTCTTTCATTACAC
*IL-6*	TTGTGCAATGGCAATTCTG	CGGACTCTGGCTTTGTCTTTC
*TNF-α*	TTCTCATTCCTGCTTGTGG	CACTTGGTGGTTTGCTACG
*IFN-γ*	TCAAGTGCGATAGATGTGGAAGAA	TGGCTCTGCAGGATTTTCATG
*CCL-2*	CAGCTCTCTCTTCCTCCACC	TGGGATCATCTTGCTGGTGA
*CCL-3*	CCAGCCAGGTGTCATTTTCC	AGGCATTCAGTTCCAGGTCA
*TLR2*	CTGAGAATGATGTGGGCGTG	TTAAAGGGCGGGTCAGAGTT
*TLR4*	TCTAACTTCCCTCCTGCGAC	ACGATCTGTAACTGGTGGCA
*MyD88*	CCATTGCCAGCGAGCTAATT	TCTGTTGGACACCTGGAGAC
*NRF2*	TCCATTTACGGAGACCCACC	GGCCGTTCTGTTTGACACTT
*HO-1*	CAGGTGTCCAGAGAAGGCTT	GCTTGTTGCGCTCTATCTCC
*GAPDH*	AAGCCCATCACCATCTTCCA	CACCAGTAGACTCCACGACA

## Data Availability

The sequencing data generated in this study have been submitted to the NCBI Sequence Read Archive (BioProject: PRJNA698466).

## Sample Availability

Samples of the compounds are not available from the authors.
